# Multiyear microgrid data from a research building in Tsukuba, Japan

**DOI:** 10.1038/sdata.2019.20

**Published:** 2019-02-19

**Authors:** Karina Vink, Eriko Ankyu, Michihisa Koyama

**Affiliations:** 1 Technology Integration Unit (TIU), Global Research Center for Environment and Energy based on Nanomaterials Science (GREEN), National Institute for Materials Science (NIMS), 1-1 Namiki, Tsukuba, Ibaraki 305-0044, Japan

**Keywords:** Energy supply and demand, Energy management

## Abstract

Microgrids comprising renewable energy technologies are often modelled and optimised from a theoretical point of view. Verification of theoretical systems with data of actually implemented systems in the field rarely occurs in an open manner, especially on the intermediate scale of research buildings. To enable modelling of the actual microgrid performance of a research environment, we present a multiyear dataset of a microgrid with solar arrays and a battery. The main energy datasets comprise data per second supplemented by hourly solar irradiation data. These may be combined with data concerning the hourly electricity prices from the main grid and the low-electricity-price periods of national holidays. The level of detail of the data per second in combination with the hourly data in these datasets allows for a comparison to the efficiency and weather-parameter correlation of other renewable energy technologies, as well as forecasting future energy generation and consumption.

## Background & Summary

The current datasets consist of data related to a microgrid equipped with renewable energy systems and are gathered by an energy management system (EMS). An EMS monitors and regulates energy input and output and can vary in scale from households, to buildings, to communities. According to ISO 50001:2011 on EMS (ISO 50001:2011 – Energy Management System, International Organization for Standardization, https://www.iso.org/iso-50001-energy-management.html, 2011), the goals of an EMS are to:

- Develop a policy for more efficient use of energy
- Fix targets and objectives to meet the policy
- Use data to better understand and make decisions about energy use
- Measure the results
- Review how well the policy works
- Continually improve energy management

Although EMS can be applied to systems without microgrids, and neither EMS nor microgrids necessarily include renewable sources of energy, since 2000, there has been an evolving trend to manage energy generation and distribution by means of smart grids^1^. These have the advantages of improved energy reliability and security, shifted peak loads, increased efficiency, and reduced greenhouse gas emissions. In a similar trend, a more sustainable and integrated outlook on energy regulation has been taken by the Japan Smart Community Alliance^2^, who states that the main goals of a smart community are:

- Enabling substantial CO_2_ reductions and energy savings
- Enabling the integration of a large amount of renewable energy
- Enabling a stabilized supply of energy
- Minimizing energy costs
- Enabling an enhanced standard of living for local residents
- Offering highly convenient transportation and water services
- Enabling the development of a safe and disaster-resistant society

These goals are much in line with the goals of the concerned microgrid, albeit that the current scale is on a building rather than community level.

Although there has been an increase in the number of studies regarding microgrids and smart grids applying renewable energy systems^3^, these often involve theoretical studies about optimisation^4–7^, whereas few studies perform field verification to test the actual system efficiency^8^, let alone on the building scale^9^. The building considered in the current study is a combined office and laboratory research building of five stories, which has been open since 2012.

Actual building energy consumption datasets have recently been published for households^10^ and office buildings^11^ that are not equipped with or connected to renewable energy systems. The dataset provided through this study contains the data per second of the building energy consumption, battery charging/discharging, and solar photovoltaic (PV) power generation; hourly data for solar irradiation; and related electricity prices per kWh. This allows for the examination of both the actual energy use of a research building with renewable energy systems, of which there are few studies published to date^9^, as well as the analysis of the efficiency of the PV and the battery.

The datasets may further be used as a basis for operational and planning studies of similar sites or for a comparison to freely available local weather data, and the values of each of the parameters may be forecast into the future. Individual array details are supplied to allow for a detailed efficiency analysis of different solar panel types. There are potential applications of these datasets in the fields of EMS design, energy-saving and demand forecasting, energy storage, computational optimization techniques and machine learning, economics and regulatory frameworks, and undoubtedly others.

## Methods

### Situational details

The individual datasets were collected from the NanoGREEN/WPI-MANA research building of the National Institute for Materials Science (NIMS). This is the first public building in Japan that has been equipped with a microgrid system consisting of four arrays of solar panels, a lead-acid battery, and an emergency backup generator (International Center for Materials Nanoarchitectonics (WPI-MANA), National Institute for Materials Science (NIMS), http://www.nims.go.jp/mana/about/building.html, 2018). In addition to this, a device is installed to measure solar irradiance. The purpose of the battery is three-fold: 1) to regulate frequency fluctuations within the microgrid, 2) to serve as backup energy supply during emergencies, and 3) to enable a peak cut of the energy demand and thereby reduce costs. The microgrid is incorporated within the Building Energy Management System (BEMS), which also records and regulates functions such as air conditioning, experimental gases, cooling and heating water, waste water, and electricity to 15 other research buildings on the same site. The microgrid was installed and tuned in 2012 and began operation on 28 March 2013. The solar arrays have received no maintenance in the form of cleaning or recalibration since their installation. The building occupancy and laboratory equipment have increased since 2012 to a point where now, in 2018, both office space and laboratories are nearly fully occupied.

### Microgrid components

**Figure 1:**
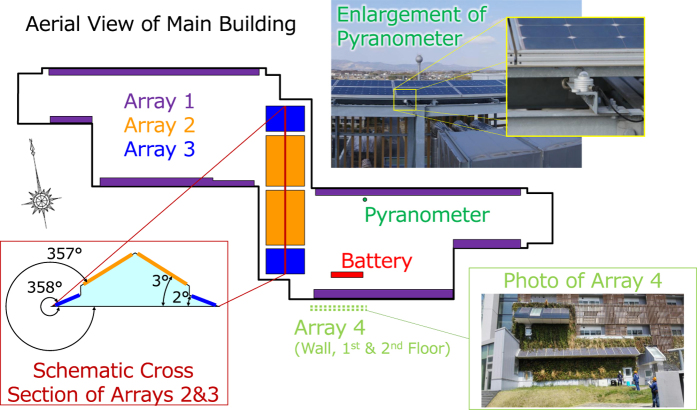
Schematic locations of the roof-mounted solar arrays as well as pictures of pyranometer and side-wall solar array.

The main components of interest in the microgrid to this study are the four arrays of solar panels, a lead-acid battery, and a pyranometer (see Fig. 1). There is also a backup power generator, which can be initiated during emergency power failures, although this has not occurred during the period of data recording. The details of the individual arrays can be found in Table 1. The azimuth of the building to which the solar arrays are mounted is 190° SW according to the building design drawing.

The lead-acid battery has a rated capacity of 326 kWh and a control maximum of 90 kW. The regular operation lies between 30 and 95% of the battery state of charge (SOC). The SOC is calibrated typically once a week, at night. The calibration is based on the voltage, which increases during charging and vice versa. When the voltage reaches the value of the specification during charging, the SOC is reset to 100%. Although the degradation of the battery may result in a different state at the same voltage, the same voltage value is used for calibration. As mentioned above, the battery has three functions: power quality assurance, backup power storage, and critical peak cut. The second function limits the minimum battery level of 30%; i.e., 30% of the capacity is saved as emergency backup. The last function is active only during the summer months (July–September), as peak demand usually occurs during those months. The functions of power quality assurance and emergency backup are active year-round and are of a higher priority than the function of critical peak cut to save both power and costs. Therefore, the operation of the battery is designed to have neither a large cost advantage nor burden for the consumer. Theoretically, the battery could serve a certain role in cost reduction due to its current operation of charging at night, when the electricity price is lower, and discharging during the peak hours of 13:00–16:00, when the price is higher. In general, the economic value of a constant emergency backup power supply is difficult to determine. The value of the lost load depends on many factors^12^, and in the setting of a research building, it is highly dependent on the type of experiments being run, the number of samples, and the treatment of the samples at the time of a blackout. Thus, calculating the value of the lost load would require additional information, which is beyond the scope of the current dataset publication.

The device to measure solar irradiance (pyranometer) is model LP PYRA 03. As indicated by the manufacturer^13^, “the measured irradiance (Global Irradiance) is the result of the sum of direct solar irradiance and of diffuse irradiance. LP PYRA 03 is a Second Class pyranometer”. We believe the values recorded by the pyranometer may be less accurate than optimally possible for two reasons.

The first reason is related to its placement. The guidelines specify that the pyranometer be placed in a clear horizontal plane, without being hindered by any buildings, construction, trees, or obstructions. If this proves impossible, the pyranometer should be placed “where obstructions in the path of the sun from sunrise to sunset do not exceed 5 degrees of elevation”, as “the presence of obstructions on the horizon line affects significantly the measurement of direct irradiance”. This includes obstructions that can reflect light or produce shadows on the pyranometer, such as solar panels. We have found that the pyranometer has been placed directly at the bottom of Array 1, which consists of solar panels angled at 10°, and is therefore obstructed from the north side (see Fig. 1).

The second reason lies in the lack of maintenance, as the guidelines recommend both cleaning the outer glass dome and annually calibrating the pyranometer to ensure its accuracy, yet neither of these has taken place over the last six years since its installation.

### Data extraction

#### Microgrid data per second

Data extraction was performed on 26 and 27 April 2018. The data per second can be extracted through an extraction program compatible with systems running on Windows 7 and a Japanese OS. This program can extract a maximum of 16 parameters at a time. The data files created in this manner were CSV files, to be opened in Excel (Office 2010 edition, with Visual Basic 6.0 installed). Due to the limitations of Excel, the maximum number of rows a single file can contain is 1,048,576, meaning that the maximum number of days per file is limited to 12 (60 s × 60 min × 24 h × 12 d = 1,036,800 rows). We have extracted all data into 119 separate files for the period from 1 January 2015 to 24 April 2018.

#### Hourly solar irradiation data

Hourly solar irradiation data are collected automatically and transferred and stored into the BEMS. These are stored in daily files at 00:01 the next day. The hourly data is stored for up to one year and one month on a local server. Two datasets were made available through Seiko Solutions, Inc., who designed and manage the microgrid. Data extraction from the BEMS was performed on 26 and 27 April 2018, which led to a dataset that runs from 1 April 2017 to 24 April 2018. This was done by connecting a computer to the BEMS and transferring the available files (.txt format) through a LAN cable. A second dataset was made available through Seiko Solutions, Inc., and ran from 1 November 2015 to 16 March 2017. When combining these two available datasets, this has unfortunately left a data gap of two weeks from 17 March 2017 to 31 March 2017 in the hourly dataset. The parameter of interest in this data was solar irradiation.

#### Hourly electricity prices

The current prices are available on the Tokyo Electric Power Company (TEPCO) Holdings website^14^. An overview of past prices was obtained through the Facility Management Office of NIMS and added to the dataset (Data Citation 1 and Data Citation 2). Note that additional fees pertaining to the entire site and therefore other buildings are not included, such as fuel regulatory costs (adjustment charges) and basic connection and spare line fees.

#### National and additional holidays

Known national holidays were taken from the cabinet office of Japan (Table 2)^15^. In case a holiday falls on a Sunday, the next weekday becomes a holiday. Note that Mountain Day, 11 August, was first observed in 2016, and the Emperor’s Birthday will change due to the scheduled abdication of the present Emperor in April 2019.

### Code availability

Extraction of the data per second requires a computer with a Japanese OS using Windows 7 and requires Visual Basic 6.0, Excel 2007/2010, and the ability to connect to the BEMS through a LAN cable. The included code files (csv_sec) need to be installed in the folder with path “C:\user” (alternatively, the configuration settings can be adapted). Running the program “createSecCsv.exe” (type: application) allows the user to specify the time period from which to extract data. Upon pressing the button “create csv file” the data is output to an Excel sheet. Thirteen parameters from the 122 total available parameters can be selected for extraction by changing the last listed values in the file “CreateSecScv.ini” (type: configuration settings) named “device number”. To enable understanding of the various parts of the code, written in Japanese, translations are given in parentheses in a separate file. Translations of the different device numbers, from which a selection of parameters can be made, are included in the dataset.

## Data Records

The datasets in this study are available through the repositories of (Data Citation 1 and Data Citation 2). An overview of the datasets is given in Table 3. The microgrid data is available in both raw and cleaned formats for data per second. In addition, the data per second has been summarised into hourly data for easy comparison. To enable understanding of the different recorded parameters, their machine codes, and Japanese labels, Table 4 provides their translation/description.

Two further datasets cover the electricity prices in Japanese Yen/kWh (Table 5), and a list of the holidays that influence the price in effect (Table 6). A final dataset consists of the code used to extract data per second from the microgrid to a stand-alone computer.

## Technical Validation

The data per second is available for a total of 1,210 days; the hourly solar irradiation data is available for two periods of 502 and 389 days each (891 days combined). We have examined the data to identify and remove erroneous data, which is inappropriate for use, resulting in the cleaned data sets. Note that the dates and times themselves have not been removed. The most voluminous data are the raw data per second, which combined have data on 12 parameters for 104,544,000 s, leading to 1,254,528,000 values in total (excluding headers).

### Known issues

- The data per second runs from 1 January 2015 to 24 April 2018. The available hourly solar irradiation data runs from 1 November 2015 to 16 March 2017, and 1 April 2017 to 24 April 2018. This leaves two gaps in the hourly solar irradiation data, during which solar irradiation is not available (from 1 January 2015 to 31 October 2015 and from 17 March 2017 to 31 March 2017).
- Maintenance occurred on the following Saturdays: 14 November 2015, 19 November 2016, and 18 November 2017. On both the maintenance and preceding days, many error values of −999,999 are recorded, meaning that the microgrid does not record appropriate data values. Note that error values of −999,999 are recorded on other days due to certain unknown errors. Those values are removed and left blank in the cleaned datasets.
- Negative values are recorded for the total active power generation by all four solar arrays during night time when solar irradiation does not occur. Those negative values are removed and set as 0 in the cleaned data. A total of 882,281 data points have been removed in this manner.
- Values of 0 are recorded for the direct voltage of the battery on the maintenance and preceding days. Those values, as well as the inappropriate data before and after those data, are removed and left blank in the cleaned data.
- Values between −680 and −450 ampere are recorded for the direct current of the battery. Those values are judged to be out of the appropriate range of operation based on the physically reasonable operational range of the direct voltage of battery and the output power of the battery. Therefore, those values are removed and left blank in the cleaned data.
- Values of 0 are recorded for the voltage of purchased electricity at the receiving end between 20:14:33 and 20:14:56 on 25 September 2017. A value of 342 volts recorded at 20:14:32 on the same day is also regarded to be inappropriate considering the typical grid voltage of 6,600 volts. Those values are removed and left blank in the cleaned data.
- Values of 0 are recorded for the active power of purchased electricity at the receiving end between 20:14:32 and 20:15:08 on 25 September 2017. Those values are removed and left blank in the cleaned data. Note that the data of the following time period starting from 20:15:09 were certain finite values, however, those appear to be inappropriate considering the trends on other days. Those are retained in the cleaned data, but should be carefully analysed before use. In addition, inappropriate data was observed on the days preceding maintenance. Apparent error values such as 0 or 2400 are recorded from 11:24:45 to 11:26:06 on 13 November 2015. Those are removed and left blank in the cleaned data. Extremely low values, for example below 200, are recorded on 18 November 2016 and 17 November 2017. Those are difficult to be distinguished from the other data and retained in the cleaned data. Therefore, those should be carefully analysed before use. Due to certain unknown factors, including battery degradation, some strange values or behaviours are occasionally recorded. A value of 0% has been recorded for the battery SOC on 19 November 2016. This is removed and left blank in the cleaned data. Between 15:03:46 and 15:03:47 on 24 February 2017, a big gap of 3.95% is recorded. Thereafter, unreasonable behaviours are recorded such as a constant SOC value lasting several days. This unreasonable behaviour ends with another big gap between 15:40:21 and 15:40:22 on 17 March 2017. Those data are removed and left blank in the cleaned data. Note that the SOC may exceed 100% during the calibration because the calibration is based on the voltage. Values higher than 100% are retained, even in the cleaned data.
- An error occurred in the hourly solar irradiation data on Friday 13 January 2017, during which no value is recorded at 16:00.

## Usage Notes

It may be necessary to convert the encoding of the CSV files to UTF-8-BOM to view Japanese characters in case the operating system does not recognize these correctly. We recommend opening the files with freely downloadable software such as Notepad++ Version 7.5.6^16^, converting the encoding (Encoding > Convert to UTF-8-BOM), and saving the file before opening in software such as Excel.

**Figure 2:**
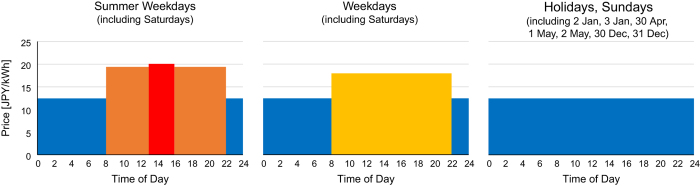
Electricity prices in 2017.

To calculate the economic efficiency of the individual solar arrays or the battery, we have included a dataset concerning the electricity price per kWh, which varies. For each hour, it is important to determine which of the four variable prices are in effect (Fig. 2). The feed-in tariff (FIT) surcharge is a required additional charge, which is always in effect during the recorded period. In addition, a solar surcharge under the renewable portfolio standards, which was in effect before the start of FIT in July 2012, was in effect until September 2014. Note that NIMS was exempted from the surcharge under renewable portfolio standards. The factor overruling all other remaining conditions is the occurrence of a Sunday or national holiday, during which the night time price is in effect. In addition, the same price applies to: 2 January, 3 January, 30 April, 1 May, 2 May, 30 December, and 31 December^16^. Next, it is important to determine the season. If it is summer, from 1 July to 30 September, two daytime prices are in effect. Between 8:00 and 13:00 and between 16:00 and 22:00, the summer daytime price is in effect. Between 13:00 and 16:00, the summer peak time price is in effect. When it is not summer, from 1 October to 30 June, the (regular) daytime price is in effect between 8:00 and 22:00. The night time price applies to all other times.

**Figure 3:**
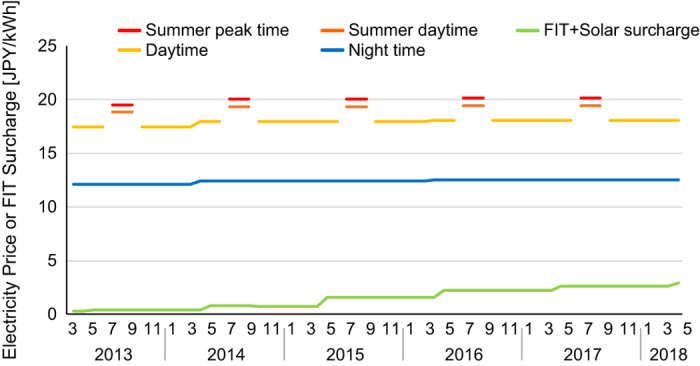
Electricity prices and surcharges from March 2013 to May 2018.

Finally, because the electricity prices change yearly as the contract is updated, it is important to determine the year. Figure 3 shows the past trend of prices. The dataset on electricity prices contains information on the entire period of operation since its initiation in 2013, to enable potential estimations of the hypothetical past and future trends.

For a comparison to local weather data, users are recommended to download the freely available hourly data measured at the Aerological Observatory in Tsukuba, situated approximately 1,500 meters away from the NanoGREEN/WPI-MANA building, from the Japan Meteorological Agency. Note that the website has a Japanese interface (Past weather data, Download (Japanese), Japan Meteorological Agency, http://www.data.jma.go.jp/gmd/risk/obsdl/index.php, 2018).

## Additional information

**How to cite this article**: Vink, K. *et al*. Multiyear microgrid data from a research building in Tsukuba, Japan. *Sci. Data*. 6:190020 https://doi.org/10.1038/sdata.2019.20 (2019).

**Publisher’s note**: Springer Nature remains neutral with regard to jurisdictional claims in published maps and institutional affiliations.

## Supplementary Material



## Figures and Tables

**Table 1:** Details of the individual arrays of solar panels.

**Array #**	**1**	**2**								**3**^a^		**4**	**Total**
Location	East and West sides, roof	Centre, roof								Centre, roof		South side, wall	
Installation type	Mounted about 3 m high	Building integrated								Building integrated		Building integrated	
Capacity (kWp)	50.92	24.74								11.40		3.78	**90.84**
Contribution to total capacity (%)	56.05	27.23								12.55		4.16	**100**
Type of crystalline silicon	mono	poly								mono		poly	
Approximate altitude angle (°)	10	3^b^ 357^b^								2^b^ 358^b^		45	
Brand	Sharp	Kyocera								Sharp		Sharp	
Module type	NQ-134LW	MSRII-PVP62AK								NUOD062-067	NUOD058-061	ND-VOL7H	
# of panels	380	200 (south)	199 (north)	36	24	18	**857**						
Panel size (m) dimension 1	1.311	1.385	1.940	1.740	1.652								
Panel size (m) dimension 2	0.668	0.455	0.9665	0.9665	0.994								
Area (m^2^) (total panels)	332.78	251.44	67.50	40.36	29.56		**721.64**						
Nominal output per panel effective surface (Wp)	133.5	62	196	178	210								
^a^The north- and south-facing parts of Array 3 each consist of 18 of the larger NUOD062-067 panels and 12 of the smaller NUOD058-061 panels.							
^b^Arrays 2 and 3 are placed on opposite sides of a triangular shaped roof, angled towards the north and south (see Fig. 1).							

**Table 2:** List of Japanese national holidays.

Name of holiday	(Approximate) date
New Year’s Day	1 January
Coming of Age Day	Second Monday of January
National Foundation Day	11 February
Vernal Equinox Day	Vernal Equinox, 20 or 21 March
Showa Day	29 April
Constitution Memorial Day	3 May
Greenery Day	4 May
Children’s Day	5 May
Marine Day	Third Monday of July
Mountain Day	11 August
Respect for the Aged Day	Third Monday of September
Autumnal Equinox Day	Autumnal Equinox, 22 or 23 September
Health and Sports Day	Second Monday of October
Culture Day	3 November
Labor Thanksgiving Day	23 November
Emperor’s Birthday	23 December

**Table 3:** Number of files and sizes in full dataset.

Data topic	# of files	Total Size	Notes
Raw, data per second (12 parameters)	119	1.3 GB	Zip file, from 1 January 2015 to 24 April 2018. Data are stored as three files per month.
Cleaned, data per second (12 parameters)	119	1.3 GB	Zip file, error values removed.
Raw, hourly solar irradiation data (1 parameter)	1	343 kB	From 1 November 2015 to 16 March 2017 and 1 April 2017 to 24 April 2018.
Summarized data per second (12 parameters)	1	3.5 MB	Converted into hourly data for easy comparison to hourly solar irradiation data.
Electricity prices	1	2 kB	
Holidays	1	1 kB	
Code for CSV extraction	6	24 kB	Zip file

**Table 4:** Translations/descriptions of the 13 provided parameters.

Machine code	Attribute	Translation/description in English	Unit
10101	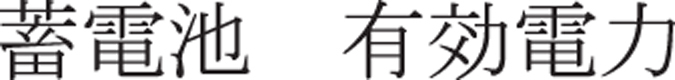	Active power of the battery	kW
10105	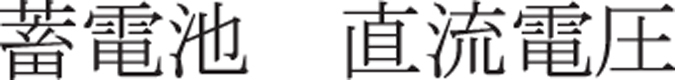	Direct voltage of the battery	V
10106	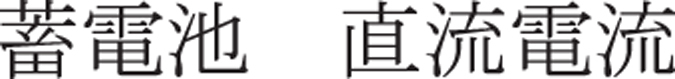	Direct current of the battery	A
10201	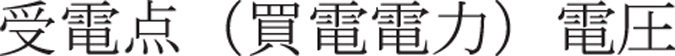	Voltage of purchased electricity at the receiving end	V
10203	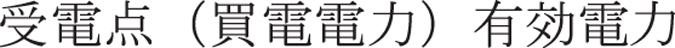	Active power of purchased electricity at the receiving end	kW
10307	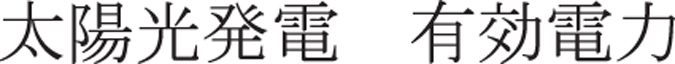	Total active power generation by all four solar arrays	kW
12144	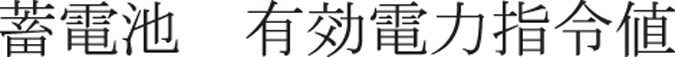	Active battery power command value	kW
12152	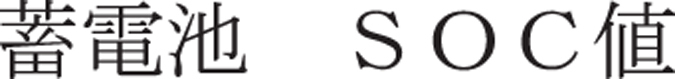	State of charge of the battery	%
20104	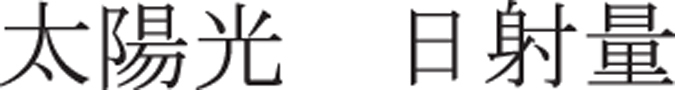	Solar irradiance	W/m^2^
20106	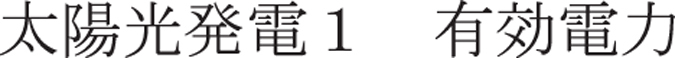	Active power generation by solar array 1	kW
20109	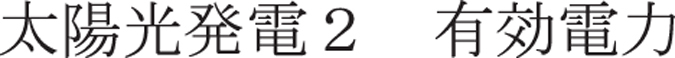	Active power generation by solar array 2	kW
20112	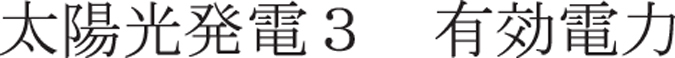	Active power generation by solar array 3	kW
20115	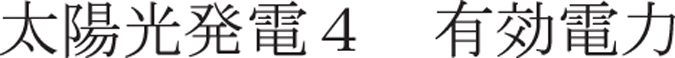	Active power generation by solar array 4	kW

**Table 5:** Dataset file metadata of electricity prices.

Attribute	Description
Period	Month and year of the price
Summer daytime	Electricity price during summer daytime
Peak time	Electricity price during summer peak time
Daytime	Electricity price during daytime
Night time	Electricity price during night time, Sundays, or holidays
FIT surcharge	Feed-in tariff (FIT) surcharge
Solar surcharge	A solar surcharge under the renewable portfolio standards

**Table 6:** Dataset file metadata of holidays.

Attribute	Description
Date	Date of Japanese national holidays
